# Own-price, cross-price, and expenditure elasticities on sugar-sweetened beverages in Guatemala

**DOI:** 10.1371/journal.pone.0205931

**Published:** 2018-10-22

**Authors:** Violeta Chacon, Guillermo Paraje, Joaquin Barnoya, Frank J. Chaloupka

**Affiliations:** 1 Cardiovascular Surgery Unit of Guatemala, Guatemala City, Guatemala; 2 Universidad Adolfo Ibáñez, Santiago, Chile; 3 American Cancer Society, Atlanta, Georgia, United States of America; 4 Washington University in St. Louis, St. Louis, Missouri, United States of America; 5 Institute for Health Research and Policy, University of Illinois at Chicago, Chicago, Illinois, United States of America; RTI International, UNITED STATES

## Abstract

**Background:**

The obesity epidemic is spreading rapidly in Guatemala, a low/middle income country still struggling with undernutrition. Sugar sweetened beverages (SSBs) consumption is strongly associated with overweight, obesity, and non-communicable diseases. In Guatemala, SSBs are readily available and consumption is high, particularly among adolescents. SSB taxes have been proposed as a cost-effective way to reduce consumption and generate revenues for public health, as has been demonstrated in several countries around the world.

**Objective:**

To estimate the price, expenditure, quality, and cross-price elasticity of beverage demand using household survey data.

**Method:**

We conducted a secondary analysis on the 2014 Guatemala Living Conditions National Survey that includes national representative household data on expenditure. Own price, expenditure, quality, and cross-price elasticities of milk, soft drinks, packaged juices, and bottled water were estimated using Deaton’s Almost Ideal Demand System (AIDS), controlling for goods’ quality. Household characteristics and beverage expenditure are summarized for urban and rural locations using descriptive statistics.

**Results:**

Positive expenditure on soft drinks was highest (50.9% of households). Positive expenditure on bottled water was next for urban households (43.8%) and lowest for rural households (10.8%). Own-price elasticities for all beverages are negative and statistically significant. Own-price elasticity of soft drinks is -1.39, suggesting that with a 10% increase in price, consumption would decrease by 13.9%. Expenditure elasticity for soft drinks (0.99) suggests that a 10% household expenditure increase would result in a 9.9% increase in demand. Milk (0.07) and soft drinks (0.07) have positive quality elasticity implying that, as household total expenditure increases, the quality of these beverages, measured by their unit values, also increases.

**Conclusion:**

Soft drink demand is highly sensitive to changes in prices, suggesting that SSB taxes could significantly reduce consumption, which, in turn, could contribute to curbing the overweight/obesity epidemic.

## Introduction

Guatemala, a lower-middle income country (LMIC), is currently struggling with the double burden of disease [1]. Childhood stunting is one of the highest in the world (49%), while overweight and obesity, and the non-communicable diseases (NCDs) related to these conditions, are rapidly rising [2]. Among other factors, sugar-sweetened beverage (SSB) consumption, is a key contributor to the obesity and NCDs epidemic [3].

There is a clear association between SSB consumption, overweight/obesity, and NCDs [3]. SSB consumption significantly increases calorie intake and body weight [4] and it is strongly associated with type 2 diabetes [5, 6], cardiovascular disease [7], and some cancers [8, 9]. SSBs consumption in Guatemala is one of the highest in Latin America (women drink 2.69 servings per day and men, 2.90, compared to the already high Central American average of 1.54 and 1.68, respectively) [10]. Annual *per capita* consumption in Guatemala is 112 liters, higher than in Costa Rica (107 liters) and the Dominican Republic (88 liters), even though Guatemala’s *per capita* GDP is 65% lower than Costa Rica’s and 48% lower than the Dominican Republic’s [11, 12]. In addition, between 2002 and 2016, total consumption of soft drinks increased 72% and *per capita* consumption increased by 27% [11]. Among carbonated beverages, those that are sugar-sweetened represent at least 68% of total consumption (soft drinks represent 77% of total beverage consumption, excluding water). Juices with added sugar represent at least 95% of total juice consumption (which represents 15% of total beverage consumption, excluding water)[11]. In 2016, sugar consumption from soft drinks was more than 173,000 tons (about 10.4 kg per person)[11].

SSB consumption is particularly high among adolescents in Guatemala City. Most, particularly those from lower socio-economic areas, have high soft drink consumption [13], and are subject to marketing efforts that target children [14]. Between 2011 and 2016, nominal soft drink prices increased by only 19%, while real *per capita* GDP grew by more than 30%, which means that soft drink affordability increased [11, 12].

International evidence shows that certain population-based interventions and policies, such as taxes, are effective in decreasing SSB consumption, as they are able to reduce its affordability and, thus, consumption [15, 16], which, in turn, may be effective in halting the overweight/obesity epidemic [17], while generating revenues for public health [18]. This type of taxation has been proposed or implemented in several countries around the world. A number of countries, such as Portugal [19], Brunei [20], Saudi Arabia [21], Thailand [22], Mexico [23], United Kingdom [24], Ireland, and South Africa [25], have implemented or are about to implement this tax. Six cities in the United States have done the same [26–28] despite persistent opposition from the beverage industry [29]. In some cases, a sustained reduction of SSBs consumption has already been observed, especially in low-income households [23].

Demand elasticities are key parameters, not only to evaluate the potential effects of taxes on SSB consumption, but also to estimate revenue, understand the potential differences between price elasticities and the effects of a tax, to assess heterogeneity among subgroups, and to predict the effect of price increases on consumption. Elasticities for SSBs have been estimated in several Latin American countries, such as Ecuador [30], Chile [31], Mexico [32], and Brazil [33]. A recent systematic review on price elasticities of SSBs in middle-income countries shows that for most of them, price elasticities are above 1 (in absolute values), meaning that a tax on SSBs would reduce consumption more than proportionally [16, 34]. There is no hard evidence of the effect of taxation on obesity prevalence (as these taxes are relatively recent and obesity changes in a population are achieved in the long-term). However, taxation has been associated with changes in BMI. It is important to note that given the complexity of measuring the effect of recently implemented taxes on weight, most of the existing evidence is based on epidemiological simulations and not empirical indicators.

Since 2002, in Guatemala, there has been an excise tax on distribution of soft drinks of 18 cents of Quetzal (GTQ) (exchange rate on December 14^th^, 2017, US$1 = GTQ7.34) (approximately 3% of the average price) per liter, and soft drinks are also subjected to the value added tax, like other goods and services [35]. In January 2018 a 20% tax on SSBs was proposed in Congress [36]. However, there is no certainty that it will be approved.

The objective of this article is to estimate SSB demand relevant parameters for Guatemala, specifically estimates of own-price, cross-price, expenditure, and quality elasticities, using household survey data. The usefulness of such estimates has been mentioned previously, but it should be stressed that these estimates are at the core of any serious attempt to *a-priori* assess the potential effect of SSB taxes on consumption and revenue. Given the common pattern of SSB consumption increases in many countries of Central America, it is expected that these estimates will also be informative for their cases.

## Materials and methods

We conducted a secondary analysis on data from the 2014 Guatemala Living Conditions National Survey (ENCOVI is its Spanish acronym). With a sample of 11,536 households, ENCOVI is nationally representative and provides data on income and expenditure, food consumption and production for self-consumption, health care access, and other socio-economic and demographic variables [37].

ENCOVI uses a probabilistic sampling design that draws from a two-stage stratification process. The first stage is the selection of Primary Sampling Units (PSUs) (groupings of households that are cartographically similar), while the second stage is the random selection of households within PSUs. We used the expansion factors to take this survey design into account in our estimates.

ENCOVI collects data on household expenditures and volumes for purchased milk, soft drinks, packaged juices, and bottled water in the previous 15 days (e.g., the question for this variable is “During the last 15 days, how much did you buy or consume and how much money did you spend on soft drinks?”). It also solicits data on amounts of these beverages obtained but not purchased (e.g., with the question: During the last 15 days, how much did you obtain without having to buy it?”), and the self-reported price that would have been paid if the item had been purchased. Beverage volume units (e.g., gallons, cups, quarts) were standardized to liters.

Unit values, used as a proxy for the price paid, are defined as the average expenditure per volume acquired (US$/lts). Because the survey does not identify the specific brand of goods acquired we grouped items in broader categories (i.e., milk, soft drinks, juice, bottled water). Expenditures are homogenized in real terms and expressed as United States dollars at December 2017. We plotted unit values in histograms and box plots in order to identify outliers. Observations with unusual unit values (i.e., three standard deviations above or below the average unit value for any category) were eliminated from the analysis (in total, 283 observations, representing 2.5% of the total sample, were discarded in this manner).

In addition, we considered socio-demographic variables, such as age, sex, literacy levels, and ethnicity of the household head, as well as the proportion of household members under 12 years old, area (urban/rural), and access to safe drinking water. Total per-capita expenditure on goods and services was also considered, as a proxy for the households’ socio-economic status. In addition, we considered a food insecurity variable, measured according to the Food and Agriculture Organization’s Latin American and Caribbean Scale of Food Security [38], as food insecurity has been positively associated with SSBs consumption [39, 40].

### Deaton’s Almost Ideal Demand System

We estimated own and cross-price, expenditure, and quality elasticities using Deaton’s Almost Ideal Demand System (AIDS). AIDS consists of a system of equations that allow the estimation of demand elasticities while controlling for differences in the goods’ qualities [41–43]. It is based on several assumptions, including that there are no price variations within small geographic areas (in this case, PSUs). Thus, observed unit value variations among households within such small geographic areas are due to differences in the quality of goods they chose. This premise requires that households are geographically close to each other and have reported expenditures for a similar period. Deaton’s AIDS has an important advantage over other estimation methods, because it provides an identification strategy to avoid endogeneity of prices by considering differences in quality choice. It is important to note that in the estimation of demand parameters, all households (i.e., those with positive expenditures and those with no expenditures) are considered, as it is assumed that they face the same unit values and, based on those, they decide whether and how much to consume.

Deaton’s AIDS estimates two equations:

$$w_{ghc}=\alpha_{g\;}^{0}+\beta_{g}^{0}{lnx}_{hc}+\gamma_{g}^{0}z_{hc}+\sum_{J=1}^{N}\theta_{gJ}{lnp}_{Jc}+(f_{gc}+u_{ghc}^{0})$$ (1)

$${lnuv}_{ghc}=\alpha_{g}^{1}+\beta_{g}^{1}{lnx}_{hc}+\gamma_{g}^{1}z_{hc}+\sum_{J=1}^{N}\psi_{gJ}{lnp}_{Jc}+u_{ghc}^{1}$$ (2)

where *w*_*ghc*_, in Eq 1, is the budget share for household *h*, located in area *c*, allocated to acquiring good *g*. *x*_*hc*_ is the total household expenditure, *p*_*jc*_ is the price of the *N* goods that the household can purchase and this is assumed not to vary within area *c*. *z*_*hc*_ is a vector of household characteristics and *f* is an error associated with the area (cluster), while *u* is an idiosyncratic error (i.e., for each household). Therefore, the budget shares each household allocates for each good depend on the household’s total budget, the inherent characteristics of the household, and the price of all goods purchased by each household. For Eq 2, *uv*_*ghc*_ is the unit value of good *g* for household *h* (in area *c*) and is a function of the same variables that affect *w*_*ghc*_ (except for the cluster level error).

The *z*_*hc*_ vector of household characteristics is composed of the natural logarithm of household size (number of persons living in the household); the household head’s sex, literacy (knows/does not know how to read and write), and ethnicity (indigenous/non-indigenous); whether there are children under 12 years old in the household; area (urban/rural); household’s access to safe drinking water; and household’s food security.

As the ENCOVI does not record prices of goods, these equations cannot be estimated directly. However, the assumption that households in a given small area are faced with the same prices allows these equations to be estimated in three stages [44]. The first stage consists of controlling for the socioeconomic characteristics of households in budget shares and unit values. The second stage consists of using the adjusted budget shares (from the previous stage) and unit values, averaged by area, to estimate measurement errors (error-in-variable models) between areas (clusters). The third stage separates the effect of quality and price. Deaton’s AIDS allows us to control for the quality of similar beverages by considering similar households with different expenditures, and quality expenditures can also be estimated. This three stage estimation provides total price elasticities and cross elasticities corrected for quality differences [42, 45]. Own-price elasticities are:

$$\varepsilon_{gj}=\theta_{gj}/w_{g}-\delta_{gj}$$ (3)

where *δ*_*gj*_ is the Kronecker delta (equal to 1 if g = j or to 0 otherwise) and budget shares are assessed at their means. The coefficient *β*^*0*^_*g*_ represents the elasticity of quantity demanded with respect to total expenditure, a proxy for income elasticity. Goods with positive income elasticities are considered “normal”, which means that demand rises with income. Goods with income elasticities over 1 are perceived as “luxury or superior” goods, while those with income elasticities below 1 are perceived as “necessities”.

The unit values of soft drinks and the other goods considered and assigned to each household correspond to the average of the unit values in the given geographical area that the household belongs to. The geographical areas in ENCOVI are small enough to assume that households are faced with the same prices of the goods under consideration.

We report elasticities for the entire population, as well as for groups by area (urban/rural). As a robustness exercise, we also estimate elasticities for groups by a measure of poverty (poor/non-poor) and safe drinking water access (not reported). Although the magnitude and signs of elasticity coefficients were reasonable (i.e., more elastic for poor households and those without safe water access), some of the standard errors were high and rendered some elasticities non-significant.

Standard errors for all elasticities were obtained by bootstrapping the sample 500 times. We conducted statistical analyses using the Stata statistical analysis software, version 13. All ENCOVI data were fully anonymized before we accessed them.

## Results

Compared to urban households, rural households have a larger size, less access to safe drinking water, more literate household heads, and a higher frequency of indigenous household heads (Table 1).

**Table 1:** Descriptive demographic variables by area, %.

	Total	CI 95%	Rural	CI 95%	Urban	CI 95%
Household size (mean)	4.8	(4.7, 4.8)	5.3	(5.2, 5.4)	4.4	(4.3, 4.4)
Proportion of households with a male head	78.5	(77.5, 79.6)	83.6	(82.4, 84.7)	74.1	(72.4, 75.8)
Proportion of households with children < 12 years old	26.7	(26.3, 27.1)	30.5	(29.9, 31.0)	23.4	(22.8, 24.0)
Proportion of households with literate head	75.6	(74.6, 76.7)	66.0	(64.5, 67.6)	83.9	(82.5, 85.2)
Proportion of households with indigenous head	36.3	(35.1, 37.6)	44.9	(43.2, 46.5)	28.9	(27.2, 30.6)
Proportion of households with safe drinking water	76.0	(74.9, 77.0)	61.5	(59.9, 63.1)	88.5	(87.3, 89.7)
Proportion of households with food insecurity^1^	78.2	(77.1, 79.2)	84.7	(83.5, 85.8)	72.6	(70.9, 74.3)

^1^Food insecurity according to the Food and Agriculture Organization Latin American and Caribbean Scale of Food Security

Rural and urban households’ expenditure on soft drinks are higher than for the rest of drinks considered, even bottled water. The highest monthly expenditure, for both rural and urban households, is on soft drinks. Mean monthly soft drink consumption among rural households is 3.9 liters, while in urban households it is 5.2 liters (Table 2).

**Table 2:** Beverage consumption by area, %.

	Total	CI 95%	Rural	CI 95%	Urban	CI 95%
Proportion of households with positive expenditure on						
Milk	30.2	(28.9, 31.4)	20.3	(19.3, 21.6)	38.7	(36.9, 40.6)
Soft drinks^1^	50.9	(49.6, 52.2)	46.9	(45.2, 48.5)	54.4	(52.5, 56.3)
Packaged juice	16.9	(15.9, 18.0)	14.4	(13.2, 15.7)	19.2	(17.7, 20.8)
Bottled water	28.5	(27.3, 29.8)	10.8	(9.9, 11.8)	43.8	(41.9, 45.7)
Household average monthly beverage quantity purchased (lts)						
Milk	2.8	(2.6, 2.9)	2.2	(1.9, 2.4)	3.3	(3.1, 3.5)
Soft drinks^1^	4.6	(4.4, 4.7)	3.9	(3.7, 4.1)	5.2	(4.9, 5.5)
Packaged juice	1.1	(1.1, 1.2)	0.8	(0.7, 0.9)	1.4	(1.3, 1.5)
Bottled water	24.1	(23.1, 25.1)	7.6	(6.8, 8.4)	38.3	(36.5, 40.0)
Household average monthly expenditure on beverages^2^						
Milk	2.68	(2.56, 2.81)	1.65	(1.49, 1.78)	3.58	(3.38, 3.77)
Soft drinks^1^	3.54	(3.42, 3.66)	2.98	(2.85, 3.13)	4.02	(3.83, 4.21)
Packaged juice	0.88	(0.83, 0.94)	0.63	(0.57, 0.69)	1.10	(1.02, 1.19)
Bottled water	2.14	(2.05, 2.23)	0.71	(0.64, 0.78)	3.38	(3.22, 3.53)
Household average monthly unit value per liter						
Milk	1.14	(1.13, 1.15)	1.00	(0.98, 1.02)	1.24	(1.22, 1.25)
Soft drinks^1^	0.93	(0.92, 0.94)	0.92	(0.92, 0.93)	0.94	(0.93, 0.94)
Packaged juice	1.10	(1.09, 1.12)	1.06	(1.04, 1.07)	1.14	(1.11, 1.17)
Bottled water	0.20	(0.19, 0.21)	0.31	(0.29, 0.33)	0.15	(0.15, 0.16)

^1^Includes regular and diet soft drinks

^2^Expenditures expressed in real US$.

Exchange rate at December 2017 was US$1 = GTQ7.34

Own-price elasticities for all beverages are negative and statistically significant. Own-price elasticity of soft drinks is -1.39, which suggests that a 10% price increase would decrease consumption by 13.9%. Milk, juices, and bottled water elasticities also suggest a consumption decrease if prices increase (Table 3). Own-price elasticity for soft drinks is significantly higher for rural households (-2.09) than for urban ones (-0.80). Cross-price elasticities for juices and bottled water with respect to soft drinks are not statistically significant. This does not necessarily mean there is no substitution, but that with the current data there is not enough statistical information to confirm the degree of substitution (Table 3).

**Table 3:** Matrix of own- and cross-price beverage price elasticity estimations and standard errors (SE)^1^ using Deaton’s AIDS by area.

	Milk	Soft drinks	Packaged juices	Bottled water
**Total**				
Milk	-1.04 (0.41)**	0.09 (0.27)	-0.15 (0.18)	0.28 (0.21)
Soft drinks	-0.42 (0.12)***	-1.39 (0.13)***	-0.02 (0.10)	0.03 (0.06)
Packaged juices	-0.56 (0.26)**	-0.36 (0.24)	-0.34 (0.17)**	-0.14 (0.12)
Bottled water	1.15 (0.26)***	0.12 (0.26)	0.25 (0.18)	-1.42 (0.15)***
**Rural households**				
Milk	0.44 (1.16)	0.39 (0.65)	-1.01 (0.45)**	0.58 (0.32)*
Soft drinks	-0.45 (0.26)*	-2.09 (0.29)***	0.05 (0.22)	0.02 (0.08)
Packaged juices	-2.28 (0.71)***	-0.36 (0.48)	0.88 (0.38)**	-0.15 (0.17)
Bottled water	0.66 (0.77)	0.34 (0.77)	0.19 (0.49)	-1.73 (0.33)***
**Urban households**				
Milk	-1.66 (0.27)***	0.04 (0.22)	0.11 (0.21)	-0.05 (0.15)
Soft drinks	-0.75 (0.23)***	-0.80 (0.17)***	-0.27 (0.16)*	0.17 (0.15)
Packaged juices	0.28 (0.24)	-0.46 (0.26)*	-0.82 (0.23)***	-0.34 (0.19)*
Bottled water	0.47 (0.24)**	0.06 (0.23)	0.36 (0.19)*	-1.17 (0.16)***

^1^Bootstrap standard errors after 500 replications

*Significant at 0.10

**Significant at 0.05

***Significant at 0.01

All beverages have positive expenditure elasticities, which implies they are all normal goods (Table 4). The highest expenditure elasticity (1.63) is for bottled water, suggesting that a 10% rise in household total expenditure, would increase demand by 16.3%, keeping everything else constant. Regarding soft drinks (0.99), a 10% household expenditure increase would increase demand by 9.9%. Milk and soft drinks’ positive quality elasticities show that, whenever household total expenditures increase, unit values of milk and soft drinks (a proxy for their quality) increase.

**Table 4:** Beverage expenditure and quality elasticity estimations and standard errors (SE)^1^.

	Expenditure	Quality
Milk	1.06 (0.11)**	0.07 (0.02)**
Soft drinks	0.99 (0.06)**	0.07 (0.02)**
Packaged juices	1.26 (0.12)**	0.04 (0.06)
Bottled water	1.63 (0.09)**	-0.08 (0.04)*

^1^Bootstrap standard errors after 500 replications

*Significant at 0.05

**Significant at 0.01

## Discussion

To the best of our knowledge, this is the first study to estimate elasticities of demand for beverages in Guatemala. Soft drinks in Guatemala are price elastic (-1.39), which means that a price increase for SSBs would have a large impact on consumption, keeping everything else constant. This price increase could be achieved by increasing the already existing tax on SSBs and would have a greater impact on rural households, which have limited access to safe drinking water and higher food insecurity. The rate at which producers pass such a tax increase to prices (pass-through) is an empirical matter. However, available evidence from other countries regarding SSB tax increases show that producers tend to fully pass through tax increases to prices (the pass-through rate is 1 or more) [23, 46, 47]. That may be reinforced by the fact that, as in other countries, SSB markets in Guatemala are oligopolistic (85% of the soft drinks market is controlled by five producers [11]) and pass-through tends to be higher [48].

Given the results obtained and the fact that the current excise tax on SSB in Guatemala is very low, it is possible that an increase in this tax would be effective not only in raising prices and decreasing consumption, but also in increasing public revenues, which, in turn, could be used to support public health interventions to improve childhood nutrition and water access (Guatemala’s laws allow for earmarking tax revenues). It would also be likely that a significant increase in taxes and prices could also result in a reduction in the incidence of NCDs and related mortality [49]. The potential increase in revenue, however, should be considered with the fact that producers will try to avoid the tax by reformulating their beverages by decreasing the amount of sugar (or substituting sugar with non-caloric sweeteners), if the tax is linked to the amount of sugar the beverage contains. Thus, the revenue returns of the tax could be less than its public health benefits.

Access to safe drinking water is a serious health policy concern in Guatemala. Almost 20% of the population does not have access to running water, especially in rural areas. Even households that have running water do not always have access to safe water, as it has been estimated that only 50% of the running water in the country is safe for drinking [50]. Packaged juices, soft drinks, and bottled water may be perceived as safer than tap water. This should not prevent public policies aimed at discouraging SSB consumption. On one hand, taxation on SSBs may incentivize households to switch to bottled water (often produced by the same companies that produce SSBs), which is a healthier option. On the other hand, revenues from SSB taxes could be earmarked to finance programs increasing access to safe drinking water, at least in areas where it is technically feasible to do this at a reasonable cost. The Mexican government is planning such a strategy with part of its SSB tax revenues, to be used to finance the introduction of free drinking water fountains in public schools and public spaces [51].

Guatemala is rural, for the most part, and almost half of the population self-identifies as indigenous [52]. Even though 53.7% of the country is poor, poverty is particularly high among the indigenous population [53]. The soft drink industry has adapted their marketing strategies to reach indigenous communities in rural Guatemala [54]. According to our findings, almost half of the households in rural areas, where most indigenous people live, report soft drink consumption. The finding that rural households are more sensitive to SSB price changes is consistent with findings from other countries like Mexico [32]). This implies that health gains from preventing SSBs consumption could be greater for these households, which often lack access to adequate health care [55].

Our study suggests that if household expenditures increase (for instance, with economic growth) the demand for soft drinks would increase almost proportionally. This is consistent with evidence from other developing countries, where demand for beverages is responsive to total expenditure changes [30, 31, 56]. This means that if taxes are to be increased in Guatemala, they should be designed in order to decrease soft drink and other SSBs affordability whenever incomes increase. A specific excise tax (monetary amount per liter) could be considered which has an automatic adjustment mechanism that could update the tax amount in line with inflation and income increases. If the government continues with the current ad-valorem tax (although increased), a periodic review of the tax should be conducted to adjust for income changes.

Our results suggest that, as household’s expenditures increases, the quality of consumed soft drinks increases moderately. This means that richer households may have higher expenditures on SSBs in part because they have higher expenditures (and expenditure elasticity is positive) and partly because they choose to buy more expensive brands. An SSB tax should consider this pattern and avoid artificially increasing the dispersion in prices, to deter households from moving to cheaper brands instead of decreasing their consumption. A specific excise tax would help to avoid such an incentive, as has been proven in the case of tobacco [57].

Guatemalan diets have become dependent on processed foods and soft drinks are now a common choice in both urban and rural areas [13, 54]. Policies and interventions to reduce soft drink consumption could include high taxes and could also consider restricting soft drink marketing (especially marketing aimed at children), implementing front-of-package labeling, and interventions to increase access to safe drinking water. Milk consumption should be encouraged to children, as it is currently low among children in Guatemala, especially those in lower socio-economic groups [58]. It is unlikely that milk could be a substitute for SSBs in this population, even though our results do not shed any light on this relationship. However, our results show that, as household expenditures increase, milk consumption increases significantly. An SSB tax policy that increases the tax burden on SSBs as income increases could decrease the price of milk in relation to SSB and could incentivize substitution of milk for SSBs. Again, complementary public policies encouraging milk consumption would help with this substitution. Given that milk is price elastic, a policy reducing its price (e.g., subsidies to producers, financed with SSB tax revenues) could encourage milk consumption among children.

Although these results are robust, they have some limitations. First, we do not consider consumption of all SSBs, only those that were reported on in the ENCOVI (i.e., soft drinks and packaged juices). Sports and energy drinks were not considered. However, their consumption is very low in Guatemala [11]. Second, we are not able to distinguish between diet or non-diet soft drinks. However, the literature indicates that non-diet soft drinks are consumed far more than diet drinks (for carbonates this is less than 10% of total volume sold in the off-trade channel) [11]. Third, we do not consider coffee, tea, yogurts, or natural juices as categories because ENCOVI does not collect data on whether these products contain added sugar. Fifth, we used household expenditure rather than income as a proxy for household material well-being. There is a large body of economic literature discussing the pros and cons of using expenditure or income as proxy measures, though, in general, expenditure is preferred as this tends to be less subject to measurement errors [45]. There is no *a priori* effect of this selection on elasticities. Sixth, even though it is assumed that there are no price variations among households located in the same PSU, such variations are likely to exist. Quality elasticities partially account for such variations. However, these elasticities reflect price variations for beverages both within households and across households of the same PSU. In this respect, it is not possible to account for actual quality variations. Seventh, the estimated elasticities are average elasticities and hold for average prices. It is possible that much higher taxes, producing large increases in prices would produce larger effects on quantities than those predicted here. In other words, it is possible that the greater the increase in prices, the higher the price elasticity (in absolute values) and, thus, the larger the effect on quantities. We do not know how elasticities change when prices change. Finally, since our estimations are not based on a complete demand system as in Zhen, et al. [59], we are not able to determine potential substitutes/complements among non-beverage food items. If there is substitution between SSB and other food containing sugar, we could be overestimating the effect of the SSB tax on actual sugar consumption. In such a case, a tax on all food items containing sugar or on sugar itself could be considered [60].

In conclusion, our results indicate that beverage taxes could significantly reduce beverage consumption in Guatemala. This public health strategy has the potential to impact lower income populations and contribute to the reduction of the obesity epidemic.
